# Criterion A of the DSM-5 Alternative Model for Personality Disorders in MMPI-2/RF Personality Disorder Scales

**DOI:** 10.3389/fpsyg.2021.735848

**Published:** 2021-11-26

**Authors:** Mark H. Waugh, Abby L. Mulay, E. Bailey Crittenden, Gina Rossi

**Affiliations:** ^1^Department of Psychology, University of Tennessee, Knoxville, TN, United States; ^2^Health Services Division, Oak Ridge National Laboratory, Oak Ridge, TN, United States; ^3^Community and Public Safety Psychiatry Division, Department of Psychiatry and Behavioral Sciences, Medical University of South Carolina, Charleston, SC, United States; ^4^Department of Psychology, University of Toledo, Toledo, OH, United States; ^5^Personality and Psychopathology Research Group, Department of Psychology, Vrije Universiteit Brussel (VUB), Brussels, Belgium

**Keywords:** alternative model for personality disorders, Minnesota Multiphasic Personality Inventory, personality disorders, level of personality functioning, personality disorder assessment, spectra scales

## Abstract

The Minnesota Multiphasic Personality Inventory (MMPI) instruments are frequently used to assess personality and psychopathology. Recent publications of personality disorder (PD) spectra scales for dimensionalized PD syndromes with MMPI instruments may advance PD assessment. To this end, we examined MMPI-Second Edition (2) and MMPI-2-Restructured Form (-RF) PD Spectra scales within the lens of a contemporary dimensional model of PDs, the alternative model for personality disorders (AMPD). The core dimension of PD, Criterion A of the AMPD or level of personality functioning (LPF), was characterized quantitatively within the PD Spectra scales. By sequentially factor analyzing the scales of the Severity Indices of Personality Problems (SIPP-118) to a common general factor of PD, an index of LPF external to the MMPI item pool was established. This LPF dimension was strongly represented across most PD Spectra scales. LPF variances within the PD Spectra scales were deconstructed using measures of general demoralization (RCdemoralization) and maladaptive personality traits indexed by the Personality Psychopathology-5 (PSY-5). Nuanced LPF and PD Spectra scale relationships were discerned. Dimensionalized Antisocial PD, Borderline PD, Dependent PD, and Paranoid PD showed meaningful association with LPF after demoralization, and maladaptive trait variances were removed. The examination of the MMPI-3 item pool reveals that the existing PD Spectra scale item sets are largely carried forward in the new edition of the MMPI. This suggests PD Spectra scale correlates, including LPF relationships, may be discernable in the newest edition of the MMPI, pending future study.

## Introduction

The Minnesota Multiphasic Personality Inventory (MMPI) instruments are widely taught (Mihura et al., 2017) and frequently used in clinical and forensic assessment (Camara et al., 2000; Wright et al., 2017). The MMPI-2 (Butcher et al., 1989) and the MMPI-2-Restructured Form (MMPI-2-RF; Ben-Porath and Tellegen, 2008) have also been translated into many languages (Friedman et al., 2014). Scales for assessing personality disorder (PD) have a strong tradition in the family of MMPI instruments. The main examples are the Morey et al. (1985); Levitt and Gotts (1995), and the Somwaru and Ben-Porath (1995) scales for the MMPI-2.

Dimensionalized PD scales recently have been developed for the MMPI-2 (Mulay et al., 2019) and the MMPI-2-RF (Sellbom et al., 2018). These dimensionalized scales for PD, termed *Spectra* scales, are receiving attention in the literature (e.g., Brown and Sellbom, 2020; Hale et al., 2020; Sellbom et al., 2020; Kremyar and Ben-Porath, 2021). This dimensionalization of PD assessment in the MMPI instruments is consistent with major initiatives in the mental health disciplines calling for dimensional models of psychopathology (Krueger et al., 2018), in PD (Hopwood et al., 2018b), and within the MMPI instruments (Sellbom, 2019). Two-dimensional classifications of PD are ascendent in the field. These are the Diagnostic and Statistical Manual of Mental Disorders-Fifth Edition (DSM-5) alternative model for PD (AMPD; American Psychiatric Association, 2013; see Hopwood et al., 2019) and the WHO International Classification of Diseases-11 (ICD-11; WHO, 2018; see Reed, 2018).

Strong interconnections between the MMPI instruments and the AMPD model are suggested by the convergences between the personality psychopathology five scales (PSY-5; Harkness et al., 1995) and the AMPD trait domains as well as other five factor model traits (e.g., Anderson et al., 2013; Sellbom, 2019). The PD Spectra scales are known to share variance with the PSY-5 scales (Sellbom et al., 2018; Mulay et al., 2019), although they are not isomorphic, and the PD Spectra scales complement and, at times, increment PSY-5 predictive assessment. Mulay et al. (2019) showed the PD Spectra scales correlated with Personality Inventory for DSM-5 (PID-5; Krueger et al., 2012) trait-facets in expectable ways and that expert content ratings of Spectra scale items converged closely with the AMPD maladaptive traits. Sellbom et al. (2018) showed the MMPI-2-RF Spectra scales largely demonstrated expectable correspondences with the PID-5.

In terms of the AMPD, PD Spectra scales have been studied almost entirely with respect to Criterion B (e.g., Sellbom et al., 2018; Mulay et al., 2019). Mapping relationships of AMPD Criterion A with the PD Spectra scales remains to be adequately investigated. The relative neglect of study of Criterion A correlates is well-known (see Sellbom, 2019; Zimmerman et al., 2019).

### The Present Study

Given interest in dimensional PD assessment, we studied relationships between MMPI-based PD Spectra scales and the two components of the DSM-5 AMPD. In the AMPD, PD is defined by Criterion A, using the level of personality functioning scale (LPFS), and by Criterion B, pathological personality traits (i.e., five trait domains based on 25 more narrow trait-facets). Criterion A is construed as a severity dimension of that which is common across PD and reflected by impairment in identity, self-direction, empathy, and intimacy functioning. Criterion B descends from the five-factor model (FFM) of personality and psychopathology (Krueger, 2019; Watson and Clark, 2020; Widiger and McCabe, 2020). Although we examined the MMPI-2 PD Spectra scales with respect to both AMPD Criterion A and B, our focus was on Criterion A. There are several reasons for this. First, Criterion A defines the presence of PD in the AMPD. Second, to our knowledge, there are no published reports of Criterion A relationships with MMPI-based scales. Third, Criterion A is relatively under-investigated in general (Zimmermann et al., 2019). Fourth, the evaluation of the MMPI Spectra scales would benefit from assessment of the proportion of Criterion A within the scales because Criterion A is the defining quality of PD within the AMPD. Criterion A was implemented with an independent index of LPF developed from the self-report severity indices for personality problems-118 (SIPP-118; Verheul et al., 2008).

Although the MMPI PD Spectra scales are dimensionalized, they are based on the traditional categorical PD syndromes such as specified in the DSM-III (American Psychiatric Association, 1980). Accordingly, to place the MMPI PD Spectra scales within the nomological net of contemporary dimensional models of PD, it is important to decompose the PD Spectra scales in terms of AMPD Criterion A and Criterion B variance.

To benchmark Criterion A, we developed a general PD index from sequential factor analyses of the facet scales of the SIPP-118. Of note, the SIPP-118 is an established measure of LPF that predates the AMPD and was also used in early validation of the LPFS (Morey et al., 2011). In this regard, Widiger et al. (2019) observed that factor analytic studies of PDs often find a general dimension of PD resembling the general factor of psychopathology (“*p*” factor”) as conceptualized within the hierarchal taxonomy of psychopathology (HiTOP) framework (Kotov et al., 2017). Our general PD or LPF index developed from the SIPP-118 can also be reckoned against severity markers from the MMPI instruments, which in effect are MMPI-based proxies for the *p* factor. To index Criterion B, we utilized the well-studied scales of the personality psychopathology five (PSY-5; Harkness et al., 1995) and note that they show strong conceptual and empirical convergences with Criterion B of the AMPD (Anderson et al., 2013. Thus, the PSY-5 we used as a proxy for Criterion B assessment.

## Materials and Methods

### Participants

Study participants were undergraduate and community volunteers who provided signed authorization of informed consent conforming to university research guidelines. The volunteers were chosen by undergraduate psychology students who received course credit in return. The initial sample (*N*=1,656) was reduced to a final pool of subjects (*N*=1,620) based on conventional MMPI-2 validity criteria as utilized by Mulay et al. (2019) and consistent with the standard described by Sellbom et al. (2018). For the MMPI-2, these were: Cannot Say <30, VRIN <80T, F and FB<110, Fp<100, L<80T, K<75T, and S<75T. We note that previous studies have scored and used MMPI-2-RF scales from administered MMPI-2 protocols cleansed with MMPI-2 criteria (Ben-Porath and Tellegen, 2008; Van der Heijden et al., 2010).

The study sample included 1,038 persons who identified as female (64%) and 582 who identified as male (36%). The average age of participants was 33.1years [standard deviation (*SD*)=14.9; range=16–82]. 65.4% of the participants were unmarried, 39.3% were full-time students, 49% reported current employment, 37.8% reported having sought psychological or psychiatric consultation at some point in their lives, and 4.9% reported current mental health treatment.

### Instruments and Target Scales

The Dutch version language version of the Minnesota Multiphasic Personality Inventory-2 (MMPI-2, Derksen et al., 2006) and the Severity Indices of Personality Problems (SIPP-118, Verheul et al., 2008) were administered. The MMPI-2 employs a dichotomous (true=1, false=2) response format, and the SIPP-118 uses a four-choice response format (1=fully disagree, 2=partly disagree, 3=partly agree, and 4=fully agree). The MMPI-2-RF scales were scored from 1,620 MMPI-2 protocols after the above-described validity criteria for the MMPI-2 were applied. The Mulay et al. (2019) MMPI-2 PD Spectra scales for Antisocial, Avoidant, Borderline, Dependent, Histrionic, Narcissistic, Obsessive-Compulsive, Paranoid, Schizoid, and Schizotypal PD were scored from the MMPI-2. The Sellbom et al. (2018) MMPI-2-RF PD Spectra scales for Avoidant, Borderline, Histrionic, Narcissistic, Paranoid, Obsessive–Compulsive, Schizoid, and Schizotypal PD were scored from the MMPI-2-RF. Although the Mulay et al. (2019) MMPI-2 Spectra scales include scales for Depressive and Somaticizing PD, they were not used in the present analyses to maintain comparable PD Spectra scale sets for both the MMPI-2 and the MMPI-2-RF instruments.

Also scored from the MMPI-2 and MMPI-2-RF were their respective PSY-5/PSY-5-r scales (Aggressiveness, Psychoticism, Disconstraint, Negative Emotionality/Neuroticism, Introversion/Low Positive Emotionality) and their respective Demoralization scale (RCdem/RCdem-r) from the Restructured Clinical Scales (Tellegen et al., 2003).

The 16-facet scales of the SIPP-118 were scored and used to develop a general factor of PD (GPD) which served as a proxy for LPF. The SIPP-118 facet scales are: Emotion Regulation, Effortful Control, Self-Respect, Stable Self-Image, Self-Reflexive Functioning, Enjoyment, Purposefulness, and Responsible Industry, Trustworthiness, Intimacy, Enduring Relationships, Feeling Recognized, Aggression Regulation, Frustration Tolerance, Cooperation, Respect).

### Analytic Strategy

Exploratory factor analysis (EFA) was used to develop an index for LPF independent from the MMPI instruments. A maximum likelihood principal factor analysis with Promax rotation was performed on the 16-facet scales of the SIPP-118. Results of the Kaiser–Meyer–Olkin (KMO) test for sampling adequacy (KMO=0.93) and Bartlett’s Test for sphericity (chi square=15,263, df 120, *p*<0.001) were successful and supported the EFA. We constructed a general factor of PD by estimating and saving factor scores from the SIPP-118 (based on factors with eigenvalues greater than one) through successive rounds of factor analysis, following the method of Hopwood et al. (2011). This process continued until a single general factor resulted. These sequential factor analyses of the SIPP-118 facet scales produced a general factor of PD (GPD) which we considered a benchmark for LPF in further analyses. The development of a GPD dimension from the SIPP-118 permitted parsing of variance in other measures relevant to PD. It should be noted that GPD conceptually is tantamount to Spearman’s (1904) general factor of intelligence (“*g*”) but extracted from the domain of PD indicators. In this way, GPD also is locatable within multivariate dimensional models of psychopathology such as HiTOP as kindred to the “*p*” factor (Widiger et al., 2019). This GPD dimension is also analogous to the general factor of personality when determined from common variance of personality traits (see Hopwood et al., 2011; Oltmanns et al., 2018).

Because it is known that reliability is sample dependent, we calculated Cronbach alpha coefficients for the SIPP-118 facet scales and the MMPI-2 and MMPI-2-RF PD Spectra scales (see Table 1). To study associations between LPF and the two sets of PD Spectra scales, Pearson correlations were computed between the SIPP-118 LPF dimension and 10 Mulay et al. (2019) MMPI-2 and the 10 Sellbom et al. (2018) MMPI-2-RF PD Spectra scales.

**Table 1:** Internal Consistency and Item Count for the SIPP-118 Facet and MMPI (−2/−2-RF) PD Spectra Scales.

	*α*		Number of items
SIPP-118 Facets			
Emotion Regulation	0.79		7
Effortful Control	0.73		7
Stable Self-image	0.78		7
Self-Reflexive Functioning	0.77		7
Enjoyment	0.76		7
Purposefulness	0.70		7
Responsible Industry	0.73		7
Trustworthiness	0.71		8
Intimacy	0.79		7
Enduring Relationships	0.71		7
Feeling Recognized	0.78		8
Aggression Regulation	0.80		8
Frustration Tolerance	0.73		8
Cooperation	0.75		8
Respect	0.65		7
Self-Respect	0.82		8
	Mulay et al. (2019)	Sellbom et al. (2018)	
	*α*	*α*	
PD Spectra Scales			
Antisocial PD	0.76	0.74	25/24
Avoidant PD	0.79	0.84	20/16
Borderline PD	0.84	0.85	28/35
Dependent PD	0.73	0.79	11/21
Histrionic PD	0.72	0.71	17/16
Narcissistic PD	0.71	0.65	15/19
Obsessive–Compulsive PD	0.56	0.67	14/12
Paranoid PD	0.75	0.60	16/21
Schizoid PD	0.63	0.69	9/15
Schizotypal PD	0.68	0.76	15/28

α, Cronbach’s alpha. SIPP-118, Severity Indices of Personality Problems; MMPI-2/−2-RF, Minnesota Multiphasic Personality Inventory-Second Edition and Restructured Form. PD, Personality Disorder. N=1,620.

Self-report measures of psychopathology are affected by response style (Sellbom and Bagby, 2010), non-specific general distress (Dohrenwend et al., 1980), and demoralization (Figueiredo, 2013). To parse demoralization from other personality construct-specific associations between LPF and PD Spectra scales, we used the MMPI-2/MMPI-2-RF RCdemoralization (RCdem) scale in partial correlational analyses.

Similarly, partial correlational analysis was used to study LPF and PD Spectra scale core associations removing shared variance with the PSY-5 scales, our proxy for AMPD Criterion B maladaptive traits. Finally, the associations between the LPF and PD Spectra scales were determined when combined effects of RCdem and the PSY-5 scales were removed statistically.

By calculating *r* to *z* values, the mean LPF correlations within the MMPI-2 and the MMPI-2-RF PD Spectra scales were found. These *r* to *z* comparative analyses for the mean LPF partial correlations were performed for (1) the two sets of PD Spectra scales with respect to each of RCdem and the PSY-5 scales, and (2) for second-order partial correlations for the two sets of PD Spectra scales when RCdem and PSY-5 joint variances were removed. Although listing results of these comparisons becomes quite detailed, they are important to deconstruct proportions of variance within the Criterion A index (LPF) with respect to the PD Spectra scales and the proxy Criterion B index of the PSY-5 scales.

The number of items from the MMPI-2 PD Spectra scales and from the MMPI-2-RF Spectra scales that are retained on the MMPI-3 was determined. This tabulation permitted speculation on potential future application of MMPI-2 and MMPI-2-RF Spectra scale relationships to the new edition of the MMPI, the MMPI-3, by virtue of substantial item retention.

## Results

### Level and Range of Psychopathology

To estimate level and range of self-reported psychopathology in the study sample, the T score mean, standard deviation (SD) and range for key MMPI-2 markers of severity were calculated. These were Infrequency (F) scale=53.83T (SD=11.82; range 33T – 104T); the Welsh Anxiety (A) scale=55.46T (SD=11.09; range 37T – 91T); Restructured Scale Demoralization (RCdem)=54.98T (SD=10.09; range 37T – 86T); Ego Strength (Es)=46.36T (SD=10.97; range 4T – 71T); PSY-5 Negative Emotionality (NEGE)=55.71T (SD=10.56; range 30T – 93T). The mean of the MMPI-2 standard clinical scales was 54.34T (mean range of 29.38T to 96T). Thus, although mean MMPI-based severity markers fell within 50–60T score band (Es is reverse scored as a severity marker), there was a wide range of self-reported psychology in this subject sample. Examining mean, SD, and range for MMPI-2-RF markers of severity showed similar levels [e.g., RCdem-r=55.55T (SD=10.23; range 37.92T to 86.27T)].

### Reliability and Item Characteristics

Table 1 shows the Cronbach alpha coefficients for the SIPP-118 facet scales and the MMPI-2 PD Spectra and the MMPI-2-RF PD Spectra scales. For the SIPP-118 facet scales, alpha coefficients range from a high of 0.82 for Self-Respect to a low of 0.65 for Respect. Of note, these are relatively brief scales (8 and 7 items, respectively) and internal consistency scale values are similar to other reports in the literature (e.g., Paap et al., 2021). The internal consistency results for the Mulay et al. (2019) and the Sellbom et al. (2018) PD Spectra scales generally resemble levels found in their development and validation studies and other reports (e.g., Kremyar and Ben-Porath, 2021).

### Determination of LPF From SIPP-118

The maximum likelihood principal axes EFA sequentially applied to the 16 SIPP-118 facet scales and resulting factor scores produced a GPD or LPF dimension after two rounds of analysis. This LPF dimension had an eigenvalue of 2.38 and accounted for 80% of the variance. Factor scores from this marker for Criterion A derived from the SIPP-118 correlated with the SIPP-118 domain scores as follows: Identity Integration *r*=0.96; Responsibility *r*=0.65; Self-Control *r*=0.88; Relational Capacities *r*=0.75; Social Concordance *r*=0.66. Although all five SIPP-118 domains correlated highly with this LPF dimension, self-identity pathology was the strongest correlation. This LPF index also correlated strongly with exemplar markers of severity from the MMPI-2 (RCdem=−0.81; *A*=−0.81; Es=0.65; *F*=−0.59) and from the MMPI-2-RF [RCdem-r=−0.81; F-r=−0.64; Emotional Internalizing Dysfunction (EIDr)=−0.77].

For illustrative purposes, a similar successive factoring procedure generating a GPD dimension was applied to the MMPI-2 PD Spectra scales and to the MMPI-2-RF PD Spectra scales. For the Mulay et al. (2019) MMPI-2 PD scales, the GPD dimension was found after two rounds of factoring (eigenvalue=1.53 and 51% of the variance). For the Sellbom et al. (2018) MMPI-2-RF PD scales, the GPD dimension also was reached by two rounds of factor analyses (eigenvalue=1.39 and 46% of variance). The GPD factor scores from the SIPP-118 (our independent marker for LPF) correlated highly with the MMPI-2 PD Spectra GPD and the MMPI-2-RF Spectra GPD factor scores (*r*=−0.74 and *r*=−0.49, respectively), but much stronger with the MMPI-2 PD Spectra scale GPD scores (*Z*=−11.78, *p*<0.000). The two MMPI-based PD Spectra scale GPD factor scores correlated very strongly (*r*=0.87). Subsequent analyses carried out with MMPI-based scales were conducted using the initial LPF Criterion A index derived from the SIPP-118 because it is independent of the MMPI-2 and MMPI-2-RF item sets.

### Zero-Order Correlations

Table 2 shows the first-order correlations between the SIPP-118 LPF dimension and the two sets of PD Spectra scales as well as with the RCdem and PSY-5 scales. Note that the correlations of LPF with psychopathological variables are generally negative because the SIPP-118 is keyed such that an elevation indicates higher level (less psychopathology) personality functioning.

**Table 2:** Correlations between SIPP-18 LPF Factor Scores and Mulay et al. (2019) and Sellbom et al. (2018) PD Spectra scales, Demoralization, and PSY-5 Maladaptive Traits.

	Mulay et al. (2019)	Sellbom et al. (2018)
Spectra Scale		
Antisocial PD	−0.35	−0.34
Avoidant PD	−0.44	−0.47
Borderline PD	−0.71	−0.78
Dependent PD	−0.61	−0.66
Histrionic PD	−0.04	0.15
Narcissistic PD	−0.35	0.15
Obsessive–Compulsive PD	−0.46	−0.66
Paranoid PD	−0.53	−0.53
Schizoid PD	−0.41	−0.21
Schizotypal PD	−0.48	−0.56
**Mean**	**−0.39 [−0.43, −0.35]**	**−0.43 [−0.47, −0.39]**
Demoralization
	RCdem	RCdem-r
	0.81	0.81
	MMPI-2 PSY-5	MMPI-2-RF PSY-5
**PSY-5 Traits**		
Negative Emotionality	−0.72	−0.69
Psychoticism	−0.53	−0.45
Aggressiveness	0.00	0.05
Disconstraint	−0.05	−0.19
Introversion/Low Positive Emotionality	0.37	−0.12
**Mean**	**−0.37 [−0.41, −0.33]**	**−0.31 [−0.35, −0.27]**

*95% confidence intervals (C.I.) are reported for mean correlation coefficients*.

LPF correlated significantly (*p*<0.01, 2-tailed) with all Mulay et al. (2019) PD Spectra scales except for Histrionic PD (HST) with LPF. The LPF and MMPI-2 PD Spectra scale mean correlation was *r*=−0.39 (note: mean of the absolute values of the correlations was 0.45). The borderline PD (BOR) scale showed the strongest correlation (*r*=−0.71).

All Sellbom et al. (2018) PD Spectra scales were significantly correlated (*p*<0.01) with LPF and yielded a mean correlation of *r*=−0.43 (the mean of the absolute values of the correlations was 0.48). The MMPI-2-RF borderline (BOR) also was the strongest association (*r*=−0.78). The respective MMPI-2 and the MMPI-2-RF PD Spectra scale mean correlations with LPF (*r*=−0.39 and *r*=−0.43) were not significantly different (*Z*=1.37, *p*<0.17).^1^

LPF and RCdem/RCdem-r were highly correlated and at the same magnitude for both the MMPI-2 and the MMPI-2-RF (*r*=−0.81).

The PSY-5 scales scored from the MMPI-2 and the revised PSY-5-r scales scored from the MMPI-2-RF showed generally similar patterns of correlation with LPF. Negative Emotionality (NEGE *r*=−0.72 and NEGE-r *r*=−0.69) and Psychoticism (PSYC *r*=−0.53 and PSYC-r *r*=−0.45) were correlated most strongly with LPF. Aggressiveness (AGGR and AGGR-r) correlations with LPF were nearly identical (*r*=0.00 and *r*=0.05). However, Disconstraint (DISC *r*=−0.05; DISC-r *r*=−0.19) and Introversion/Low Positive Emotionality (INTR *r*=−0.37; INTR-r *r*=−0.12) significantly differed in their correlation with LPF across the two MMPI instruments (*Z*=−4.05, *p*<0.0001; and *Z*=−6.22, *p*<0.000, respectively). The MMPI-2 INTR showed a stronger correlation with LPF than its counterpart RF version, and the MMPI-2-RF DISC-r was more strongly correlated with LPF than its counterpart −2 version.

The mean correlation of LPF with the MMPI-2 PSY-5 scales was *r*=−0.37 (mean of absolute values *r*=0.38). The mean correlation of LPF with the MMPI-2-RF PSY-5-*r* scales was *r*=−0.31 (mean of absolute values *r*=0.33). The mean correlation of LPF with the PSY-5 scales was moderately but significantly higher for the MMPI-2 than the mean correlation of the PSY-5-r scales with LPF scored from the MMPI-2-RF (*Z*=2.06, *p*<0.04).^2^

Regarding specific MMPI-2 associations, there was no difference between the mean Mulay et al. (2019) PD Spectra scale correlation with LPF (*r*=−0.39) relative to the mean MMPI-2 PSY-5 correlation with LPF (*r*=−0.37; *Z*=−0.66, *p*<0.51). The correlation between Criterion A and the Mulay et al. (2019) Spectra scales is at the same level as the correlation with Criterion B.

However, for the specific MMPI-2-RF comparisons, the mean Sellbom et al. (2018) PD Spectra scale correlation with LPF (*r*=−0.43) was significantly greater than the mean PSY-5-r correlation with LPF (*r*=−0.31; *Z*=3.96, *p*<0.0001). Thus, the Sellbom et al. (2018) Spectra scales correlate higher with Criterion A than with Criterion B defined as the PSY-5-r scales.

Of note, the convergent correlations of PD Spectra scales (e.g., MMPI-2 BOR with MMPI-2-RF BOR, etc.) across the MMPI-2 and the MMPI-2-RF were very strong, ranging from a high of 0.91 for Avoidant PD (AVD) to a low of 0.42 for Obsessive–Compulsive PD (OCPD), with a mean *r*=0.81. Convergent correlations of similar high magnitude were reported in Mulay et al. (2019) from separate large samples.

### LPF and PD Spectra Scale Associations Controlling for Demoralization

Removing the influence of RCdem/RCdem-*r* in analyses of the MMPI-2 and the MMPI-2-RF PD Spectra scale correlations with LPF, the paranoid (PAR), BOR, and antisocial (ANT) PD Spectra scales retained strong first-order partial correlation with LPF (all correlations round to −0.30). The Mulay et al. (2019) Spectra scales showed a modestly stronger and significantly different mean partial correlation with LPF relative to that for the Sellbom et al. (2018) scales (*r*=−0.20 and−0.12, respectively; *Z*=−2.27, *p*<0.02). The means of the absolute values for these respective sets of correlations was *r*=0.20 and 0.19, however, which was not different (*Z*=0.27, *p*<0.78; see Table 3).

**Table 3:** Partial correlations between SIPP-18 LPF Factor Score and Mulay et al. (2019) and Sellbom et al. (2018) PD Spectra scales controlling for Demoralization (RCdem) and Maladaptive Personality Traits (PSY-5).

Mulay et al. (2019)			
	Remove Demoralization	Remove PD Traits	Remove Both
Spectra Scale			
Antisocial PD	−0.31	−0.17	−0.17
Avoidant PD	−0.11	−0.10	−0.09
Borderline PD	−0.35	−0.21	−0.16
Dependent PD	−0.19	−0.35	−0.21
Histrionic PD	−0.09	−0.27	−0.12
Narcissistic PD	0.01	0.15	0.09
Obsessive–Compulsive PD	−0.24	−0.17	−0.12
Paranoid PD	−0.30	−14	−0.15
Schizoid PD	−0.14	−0.10	−0.10
Schizotypal PD	−0.21	−0.05	−0.02
**Mean**	**−0.19[−0.24, −0.14]**	**−0.14 [−0.19, −0.09]**	**−0.12 [−0.17, −0.07]**
Sellbom et al. (2018)			
Spectra Scale			
Antisocial PD	−0.28	−0.16	−0.12
Avoidant PD	−0.10	−0.19	−0.12
Borderline PD	−0.35	−0.47	−0.35
Dependent PD	−0.19	−0.40	−0.29
Histrionic PD	−0.09	−0.02	−0.02
Narcissistic PD	−0.13	0.14	0.03
Obsessive–Compulsive PD	−0.22	−0.17	−0.18
Paranoid PD	−0.30	−0.20	−0.17
Schizoid PD	−0.06	−0.09	−0.10
Schizotypal PD	−0.23	−0.22	−0.16
**Mean**	**−0.12 [−0.17, −0.07]**	**−0.15 [−0.20, −0.10]**	**−0.16 [−0.21, −0.11**

*95% confidence intervals (C.I.) are reported for mean correlation coefficients*.

### LPF and PD Spectra Scale Associations Controlling for PSY-5 Scales

Removing variance associated with the Criterion B maladaptive personality traits (i.e., indexed by the PSY-5 scales as a proxy for Criterion B), first-order partial correlations between the LPF and the PD Spectra scales generally decreased in strength. The mean partial correlations for the MMPI-2 and the MMPI-2-RF PD Spectra scales were not significantly different (*r*=−0.14 and−0.15; *Z*=0.23, *p*<0.82). Note that the means of the absolute values of these correlations were slightly larger (*r*=0.17 and 0.22, respectively; *Z*=−1.39, *p*<0.16). The most robust associations were found for BOR (*r*=−0.21 and−0.47) and Dependent PD (DEPN; *r*=−0.35 and−0.40). Of note, the HST PD Spectra scale from the MMPI-2 (*r*=−0.27) and the Obsessive–Compulsive PD (OCPD) Spectra scale from the MMPI-2-RF (*r*=−0.30) also showed strong associations with LPF after PSY-5 scale variances were removed.

### LPF and PD Spectra Scale Associations Controlling for Demoralization and PSY-5 Scales

Removing combined variance associated with both RCdem and the PSY-5 scales, second-order partial correlations between LPF and the two sets of PD Spectra scales were found to be generally lower. The mean partial correlation for the Mulay et al. (2019) and for the Sellbom et al. (2018) Spectra scales (*r*=−0.12 and−0.16, respectively) were not significantly different (*Z*=0.98, *p*<0.32). For the MMPI-2 PD Spectra scales, the sole relatively appreciable association was for DEPN (*r*=−0.21), while BOR (*r*=−0.16), ANT (*r*=−0.17), and PAR (*r*=−0.15) showed a degree of relevant association. With the MMPI-2-RF PD Spectra scales, moderate-level partial correlations with LPF for BOR (*r*=−0.35) and DEPN (*r*=−0.29) were found, with less robust associations for ANT (*r*=−0.12), PAR (*r*=−0.17), OCPD (*r*=−0.18) and Schizotypal PD (SZT; *r*=−0.16).

### PD Spectra Scale Items on the MMPI-3

The mean percentage of Mulay et al. (2019) PD Spectra scale items represented on the Minnesota Multiphasic Personality Inventory-Third Edition (MMPI-3; Ben-Porath and Tellegen, 2020) is 63%. SZT is the highest at nearly 87%, and OCPD is represented weakly at 36%. For the Sellbom et al. (2018) PD Spectra scales, 83% of the MMPI-2-RF items are found on the MMPI-3 (see Table 4). AVD PD is most highly represented on the MMPI-3 at 94%, and narcissistic PD (NPD) is the lowest at 74%. However, it should be noted that the Mulay et al. (2019) PD Spectra scales can be scored with the reduced set of items on the MMPI-2-RF item pool (see Mulay et al., 2019 for scoring key). The Mulay et al. (2019) PD Spectra scales scored on the MMPI-2-RF are well represented on the MMPI-3 with 89% of the original PD Spectra scale items scored on the MMPI-2-RF carried over to the MMPI-3.

**Table 4:** Personality Disorder Spectra Scales Items Carried on to the MMPI-3.

	Mulay et al. (2019) MMPI-2		Sellbom et al. (2018) MMPI-2-RF		Mulay et al. (2019) MMPI-2 to MMPI-2-RF	
	# Items Retained	%	# Items Retained	%	# Items Retained	%
Antisocial	20/25	80	21/25	84	20	100
Avoidant	9/19	47	15/16	94	8	80
Borderline	17/28	61	31/35	89	18	95
Dependent	5/11	46	16/21	76	5	83
Depressive	20/30	67	–	–	20	95
Histrionic	10/17	59	13/16	81	10	77
Narcissistic	12/15	80	14/19	74	12	92
OCPD	5/14	36	9/12	75	5	63
Paranoid	10/16	63	19/21	91	10	100
Schizoid	5/9	56	13/15	87	5	100
Schizotypal	13/15	87	23/28	82	13	93
Somaticizing	15/21	71	–	–	15	94

## Discussion

Using a well-established measure of severity of personality functioning which also contributed to validation of the Criterion A of the AMPD (Morey et al., 2011), we created an LPF index from the SIPP-118. This index was used to interrogate the PD Spectra scales from the MMPI-2 and the MMPI-2-RF with respect to the AMPD. The two sets of PD Spectra scales evidenced meaningful associations with LPF (mean absolute value correlations of 0.45 and 0.48, respectively). Notably, for both sets of PD Spectra scales, BOR, DEPN, and PAR yielded correlations with LPF greater than 0.50, with BOR manifesting the strongest relationship (correlations of 0.71 and 0.78). This result is consistent with Sharp et al. (2015) who found that a general factor of PD was highly associated with Borderline PD (BPD) and resembled Criterion A of the AMPD.

We represented Criterion B by the MMPI-2 PSY-5 and the MMPI-2-RF PSY-5-r scales (Anderson et al., 2013). We found that on average LPF also correlated strongly and significantly with these proxy Criterion B domains. Interestingly, we note that the mean Criterion A and B scale correlations in our results (*r*=−0.37 and−0.31; absolute value correlations 0.38 and 0.33; joint mean 0.35) are slightly lower than mean Criterion A and B scale associations reported by other investigators. For example, Sleep et al. (2019) reported a mean Criterion A and B correlation of.45 [using the level of personality functioning scale-self report (LPFS-SR; Morey, 2017) and the PID-5]. Hopwood et al. (2018a) similarly reported strong correlations between the LPFS-SR and numerous self-report measures of five factor model personality and maladaptive personality traits including the PID-5 (converging generally at *r* =>0.50 to 0.40). Of note, the difference between our LPF and PSY-5 mean of.35 is meaningfully different from the approximate Criterion A and B correlation of 0.45 from these comparison studies [Sleep et al., 2019 *Z*=−1.91, *p*<0.06; Hopwood et al. (2018a) *Z*=−3.56, *p*<0.0004].

The implication of this finding is unclear. Sample differences in part may be in play. Sleep et al. (2019) used adult English-speaking Amazon Mechanical Turk volunteers (*N*=308) and Hopwood et al. (2018a) studied adult English-speaking Amazon Mechanical Turk workers (*N*=1,976), whereas our sample included a mix of adult Dutch-speaking student and community volunteers of a range of nationalities (*N*=1,620). In addition, our study relied on the PSY-5 and PSY-5-r scales to represent Criterion B, rather than the PID-5 or other instruments. Anderson et al. (2013) reported counterpart correlations (range 0.44 to 0.67; Mdn 0.53) between PSY-5-r and PID-5 domain scales. Thus, the PSY-5 scales are reasonable indicators of Criterion B domains. Based on our results, it appears that the PSY-5 maladaptive trait domains carry relatively less Criterion A variance than some investigators have found with other instruments assessing Criterion B.

From partial correlational analyses, we found that removing demoralization lowered the general association between LPF and the PD Spectra scales. Nonetheless, meaningful associations remained (*r*=0.20 and 0.19 for absolute values). Thus, the non-specific influence of demoralization does not fully explain the connection between LPF and the PD Spectra scales. We deduce that the overall LPF and PD Spectra scale association reflects impaired personality functioning (i.e., LPF), a more personality-specific severity dimension than general psychiatric distress *per se*. This finding is consistent with results from (Garcia et al., 2021) who identified separable dimensions of variance for substantive LPF, psychosocial impairment, and maladaptive trait severity in indices which formed the conceptual basis for the LPFS of Criterion A. Regarding MMPI-based assessment of PD, the PD Spectra scales of ANT, BOR, and PAR yielded appreciable LPF correlations when demoralization was removed. These dimensionalized PD syndrome scales therefore evidence moderate levels of Criterion A impairment beyond general distress.

Statistically removing variance associated with the maladaptive traits of the PSY-5 scales, the two sets of PD Spectra scales continued to show meaningful association with LPF (with mean absolute value correlation of *r*=0.17 and 0.22). In other words, after parsing Criterion B trait variance from the PD Spectra scales, appreciable Criterion A variance remains. Controlling for maladaptive traits, the PD Spectra scales of BOR and especially DEPN show appreciable amounts of Criterion A. That the PD Spectra scale DEPN manifests LPF association beyond that of PSY-5 maladaptive traits suggests both the importance of the Dependent PD syndrome (Bornstein, 1993) and that the existing domains and facets of the AMPD may not adequately cover psychopathological dependency. Importantly, the PD Spectra scale DEPN continued to show robust association with LPF when both demoralization and maladaptive trait variance were removed.

Removing both demoralization and Criterion B variance, the general association between LPF and the Spectra scales (mean of absolute value of correlations) was reduced to.12 and 0.16, respectively for the MMPI-2 and MMPI-2-RF. Although these correlations are relatively low in magnitude, this finding nonetheless establishes that a degree of LPF saturation exists within the PD Spectra scales even when the influence of general distress and Criterion B are removed. The PD Spectra scales include some specific-Criterion A variance.

This AMPD analysis of MMPI-based PD Spectra raises theoretical issues about how to conceptualize PD. Although the Spectra scales are dimensionalized, they nonetheless are based on the syndrome concept of psychopathology. Contemporary approaches to psychopathology (Krueger et al., 2018), PD (Hopwood et al., 2018b), and reformulations of the MMPI scales (Sellbom, 2019) are based on hierarchal models of psychopathology with the well-known FFM a prototype Moreover, Clark and Watson (2019) modern articulation of construct validity states that measures should be adjudicated within hierarchical models of personality or psychopathology. Using the terms of Clark and Watson (2019), our results suggest the PD Spectra scales are *interstitial* if not *conglomerate* constructs. Interstitiality refers to items simultaneously sharing both important unidimensional variance (e.g., LPF) and separate but correlated dimensions (e.g., negative affectivity and detachment). For example, Avoidant and Dependent PD would share negative affectivity and detachment associations. Conglomerate constructs are “winning combinations” (Clark and Watson, 2019, p. 1414) of two or more moderately correlated dimensions that reflect a larger construct which by tradition or preference has traction in the field. The PD Spectra scales would seem to be both interstitial and conglomerate constructs. Clinical tradition and the history of syndrome concepts in psychiatry impart “conglomerate” status to the scales. Thus, the paradigmatic tensions between traditional and contemporary PD conceptions remain within the PD Spectra scales. Clark and Watson (2019) argue that the burden falls to conglomerate constructs to show they are more than linear combinations of constituent elements. However, the PD Spectra scales have points of utility.

First, traditional PD syndromes continue to be widely taught and used in clinical settings. Second, they are PD syndromes (and the PD Spectra scales) are largely decomposable with the AMPD. This permits a cross-walking of syndrome and dimensional approaches and can thus foster communication between clinicians who may draw on these different models. Third, the robust association of DEPN with LPF after demoralization and maladaptive trait variances are removed suggests that Dependent PD is not adequately specified with the AMPD. There is extensive empirical support and clinical lore associated with Dependent PD (Bornstein, 1993). MMPI-based assessment with the DEPN scale thus brings further and richer specification to the clinical evaluation of PD. To a lesser extent, the specific associations of ANT, BOR, and PAR Spectra scales with LPF also contribute important information in the clinical evaluation of PD.

Elaborating on this idea, it is important to recognize the value of appraising assessment instruments in terms of clinical utility as opposed to using psychometric purity as a gold standard. Clinical utility may be evaluated in terms of ease of use, communication value, and implications for treatment (Mullins-Sweatt and Widiger, 2009). The MMPI-based PD Spectra scales would seem to possess favorable clinical utility. The PD Spectra scales are scoreable from a widely used instrument and they were developed referencing well-known PD syndromes. Thus, they qualify for the ease of use and communication value criteria. To the extent they provide for greater specification of PD such as with the DEPN scale and that they generally embody inherent Criterion A variance, the Spectra scales can be expected to aid careful differential diagnosis of PD. Assessment of Criterion A informs psychotherapy and treatment planning of PD (Clarkin et al., 2015). For example, lower levels of LPF suggest the importance of structuring and safety-enhancing interventions. Our analyses show that the PD Spectra scales are saturated with LPF variance in addition to the correlated dimensions of demoralization and maladaptive traits. Thus, a finding of PD Spectra scale elevations increases the likelihood of PD and the clinician thereby is alerted to important treatment planning implications.

Speculating on the future of MMPI-based PD assessment, tabulating the common items from the RF-version of the Mulay et al. (2019) and the Sellbom et al. (2018) PD Spectra scales suggests that the scales may offer a platform for developing PD spectra scales with the new MMPI-3 (Ben-Porath and Tellegen, 2020). Of note, 85% of the combined RF items (of both the Mulay et al., 2019 and Sellbom et al., 2018 RF-based items) are retained on the MMPI-3. This is a strategic core item pool from which MMPI-3 PD Spectra scales could be fashioned. To do so would take advantage of the new norms and may make use of some of the new items on the MMPI-3.

### Limitations and Future Directions

Our study does have some limitations. This includes reliance on a mainly non-clinical sample. That said, it is known that the prevalence of PD is non-trivial in community (non-clinical) populations (e.g., approximately 10–11%; Lenzenweger, 2008; Winsper et al., 2020). Thus, our sample, which includes both college student and community persons, can be expected to provide some representation of psychopathology and PD. The evidence for this is suggested by the range of scores from the MMPI-2. For example, the MMPI-2 infrequency scale (F) ranged from 33 to 104T [mean=53.83 (SD=11.82)]. Similar descriptive statistics also obtained for other MMPI severity indicators. Of note, approximately 38% of the sample reported previously having received mental health treatment. Thus, our study population included a range of self-reported psychopathology even if not drawn specifically from a clinical setting. Another relative limitation of the study is the large number of zero order and partial correlations calculated. Mitigating concern about capitalizing on chance through many correlations is our large sample size (N=1,620). Criterion B was assessed by the PSY-5 scales, but there is a degree of overlap between PSY-5 and PD Spectra scale items. Measurement of maladaptive traits by a non-MMPI-based instrument such as the PID-5 would have permitted assessment of both Criterion A and B separately from the parent instrument of the PD Spectra scales. Future studies of the PD Spectra scales with respect to contemporary PD models such as the AMPD should consider this strategy.

A promising direction for future study would be to build PD Spectra scales with the MMPI-3 item pool. A foundation for this exists in that 85% of the RF-based Mulay et al. (2019) and Sellbom et al. (2018) Spectra scale items are carried over onto the MMPI-3. With the updated population norms for the MMPI-3 and addition of item content that may greater represent grandiosity and compulsivity [e.g., Self-Importance (SFI) and Compulsivity (CMP)], there is reason to expect potential MMPI-3-based Spectra scales could offer advantages in the clinical assessment of PD. Part of this advantage is the likelihood that Criterion A will be well-represented within such new scales.

In closing, our study of relationships between an LPF index and MMPI-based PD assessment suggests broad observations about Criterion A. Although some studies discuss the not insignificant empirical covariation between measures of Criterion A and B (e.g., Morey, 2019; Sleep et al., 2019), the AMPD and the ICD-11 nonetheless conceptually require a threshold assessment for presence of PD for a PD diagnosis. Because Criterion A is part of the AMPD landscape, understanding relationships with major assessment instruments for PD is clinically relevant and practical. More broadly, Criterion A can be regarded as a way to benchmark the classic construct of psychological structure or level of personality organization (Kernberg, 1967), while also capturing agentic qualities of personhood beyond stylistic expression of Criterion B maladaptive traits (Sharp and Wall, 2021). Criterion A has been found to be generally reliable, useful, and intuitive to many (see Birkhölzer et al., 2021)In a manner similar to our approach with the SIPP-118 LPF index and MMPI-based PD scales, Garcia et al. (2021) deconstructed sources of variance within several measures conceptually related to LPF. They concluded that LPF is a theoretically substantive dimension of PD severity beyond general psychiatric impairment or severity of maladaptive traits. In this regard, we side with Kurt Lewin (1943) who stated in various ways the idea that there is nothing more practical than a good theory. In assessing personality functioning and PD, Criterion A offers a theoretical base for clinical formulation. The theoretical base of Criterion A also extends to MMPI-based assessment of PD.

## Data Availability Statement

The original contributions presented in the study are included in the article, further inquiries can be directed to the corresponding author.

## Ethics Statement

Ethical review and approval was not required for the study on human participants in accordance with the local legislation and institutional requirements. The participants provided their written informed consent to participate in this study. The written informed consent form received approval from the Data Protection Office of the Vrije Universiteit Brussel (VUB), Belgium. The study followed the principles of the Declaration of Helsinki.

## Author Contributions

MW, AM, and GR contributed to the design and analyses of the study. GR was responsible for acquiring the data set. MW was lead in composing the manuscript and all authors participated in the write up. EC contributed technical work for analyses and manuscript display of results. All authors are accountable for the content of the work.

## Conflict of Interest

The authors declare that the research was conducted in the absence of any commercial or financial relationships that could be construed as a potential conflict of interest.

## Publisher’s Note

All claims expressed in this article are solely those of the authors and do not necessarily represent those of their affiliated organizations, or those of the publisher, the editors and the reviewers. Any product that may be evaluated in this article, or claim that may be made by its manufacturer, is not guaranteed or endorsed by the publisher.

## Footnotes

^1^The mean of the absolute values of each of the MMPI-2 and MMPI-2-RF PD Spectra correlations with LPF was *r* = 0.45 and 0.48, respectively, also not significantly different (*Z* = 1.05, *p* < 0.29).

^2^However, the mean of the absolute values of the MMPI-2 PSY-5 and the MMPI-2-RF PSY-5-r correlations with LPF were r = 0.38 and 0.33, respectively, not significantly different (*Z* = 1.72, *p* < 0.09).
